# hs-CRP as a Marker of Systemic Low-Grade Inflammation Is Not Associated with Steatotic Liver Disease in Adolescents: Insights from the EVA4YOU Study

**DOI:** 10.3390/metabo16020108

**Published:** 2026-02-03

**Authors:** Johannes Nairz, Alex Messner, Sophia Zollner-Kiechl, Ursula Kiechl-Kohlendorfer, Michael Knoflach

**Affiliations:** 1 VASCage, Centre on Clinical Stroke Research, 6020 Innsbruck, Austria; 2 Department of Pediatrics III, Medical University of Innsbruck, 6020 Innsbruck, Austria; 3 Department of Pediatrics II, Medical University of Innsbruck, 6020 Innsbruck, Austria; 4 Department of Neurology, Hochzirl Hospital, 6170 Zirl, Austria; 5 Department of Neurology, Medical University of Innsbruck, 6020 Innsbruck, Austria

**Keywords:** adolescents, hepatic steatosis, steatohepatitis, hs-CRP, low-grade inflammation, metabolic syndrome, obesity, dyslipidemia

## Abstract

**Objectives**: Systemic low-grade inflammation is associated with steatohepatitis in adults. We aim to explore if systemic low-grade inflammation, measured by plasma high-sensitivity C-reactive protein (hs-CRP), is also linked to steatotic liver disease in adolescents. **Methods**: In the cross-sectional Early Vascular Ageing in the YOUth study, systemic low-grade inflammation was measured by hs-CRP and liver fat content was quantified by the controlled attenuation parameters (CAP) derived from FibroScan^®^ (Echosense, Paris, France) measurements in 14- to 19-year-old Austrian adolescents. Cardiovascular risk factors and anthropometric data were collected through face-to-face interviews, physical examinations, and comprehensive fasting blood analyses. Linear regression models were performed to analyze the association between hs-CRP and CAP values. **Results**: A total of 1300 adolescents (64.6% female) with a mean age of 17.2 ± 1.3 years were included in this analysis. hs-CRP was significantly associated with CAP values in the simple linear regression model (b = 1.35, *p* = 0.044) and after adjustment for sex and age (b = 1.84, *p* = 0.006), suggesting an increase in systemic low-grade inflammation with increasing liver fat content. However, further adjustment for major factors of the metabolic syndrome (Homeostatic Model Assessment for Insulin Resistance, non-high-density lipoprotein cholesterol, body mass index z-score, systolic blood pressure z-score) led to a loss of significance of the mentioned association (b = −0.55, *p* = 0.419). **Conclusions**: Systemic low-grade inflammation measured by hs-CRP is linked to higher liver fat content in our adolescent cohort. However, this association is largely driven by components of the metabolic syndrome and the overall metabolic milieu, rather than reflecting liver-specific inflammation.

## 1. Introduction

Steatotic liver disease (SLD) refers to excessive fat accumulation in hepatocytes, which in adolescents mostly occurs in the context of metabolic dysfunction [1] and, according to the current consensus terminology, when the established diagnostic criteria are fulfilled (that is, SLD in the presence of one or more cardiometabolic risk factor(s) and the absence of harmful alcohol intake) is then referred to as ‘metabolic dysfunction-associated steatotic liver disease’ (MASLD) [2]. It is the most common chronic liver disease in pediatric patients, affecting 4.8% (Europe) up to 30% (USA) of adolescents [3,4]. The pathological course of MASLD, through inflammation, oxidative stress and abnormalities in fat metabolism, leads from simple steatosis to steatohepatitis and subsequently to liver fibrosis and cirrhosis, which potentially can even increase the risk of hepatocellular carcinoma [5,6,7]. Thus, in patients with MASLD, persistent hepatic inflammation is a key factor in the progression of liver fibrosis [8].

High-sensitivity C-reactive protein (hs-CRP) is a non-specific inflammatory marker that, within certain thresholds, indicates the presence of low-grade inflammation in the body [9,10]. It is a well-established biomarker for systemic low-grade inflammation associated with the metabolic syndrome and its components in adolescents and has been linked to the development of cardiovascular disease [11,12,13,14,15,16]. Several studies have shown that among adults, elevated hs-CRP levels also correlate positively with the severity of SLD and its risk of progression to steatohepatitis and liver fibrosis, suggesting that inflammatory responses play a key role in this regard [17,18,19,20]. So far, there are no studies analyzing the association between hs-CRP and the extent of liver steatosis in adolescents.

The aim of this study was to investigate the association between increased liver fat content, assessed non-invasively by the controlled attenuation parameter (CAP) using FibroScan^®^ (Echosense, Paris, France), and hs-CRP as a marker of systemic low-grade inflammation in our adolescent Early Vascular Ageing in the YOUth (EVA4YOU) study cohort. We further adjusted our models for components of the metabolic syndrome to assess whether any observed association was driven by the metabolic milieu rather than liver-specific inflammation.

## 2. Materials and Methods

### 2.1. Study Population

This analysis is based on data from the EVA4YOU study (www.clinicaltrials.gov; Identifier: NCT04598685; date of registration: 15 October 2020), a single-center cross-sectional study conducted between January 2021 and March 2023 in Tyrol, Austria—a federal state in western Austria with approximately 760,000 inhabitants. The study aimed to assess cardiovascular health and early manifestation of vascular ageing in adolescents aged 14 to 19 years.

A total of 1517 adolescents were recruited through 57 participating schools and companies all over Tyrol. Data collection was performed on-site by specially trained medical staff and included, among other assessments, a structured interview, blood sampling, liver fat quantification, and anthropometric measurements. All data were recorded using an electronic case report form.

The study was approved by the ethics committee of the Medical University of Innsbruck (approval number: 1053/2020) and was conducted in accordance with the Declaration of Helsinki. Written informed consent was obtained from all participants, and for minors, additionally from their legal guardians. There were no exclusion criteria apart from missing informed consent or absence on the day of examination.

For the present analysis, only participants with complete and valid FibroScan^®^ measurements, complete blood samples, and without unspecific hs-CRP elevation (≥10.0 mg/L) were included.

### 2.2. Laboratory Analysis

Blood samples were collected in the morning after an overnight fast, cooled, and immediately transported to the Central Institute of Medical and Chemical Laboratory Diagnostics at the University Hospital Innsbruck, Austria. hs-CRP was assessed with a particle-enhanced immunological clouding assay, whereby hs-CRP levels ≥ 10.0 mg/L were classified as unspecific elevation in the context of a possible acute infection and therefore excluded from the analysis regarding systemic low-grade inflammation [21,22].

Plasma total cholesterol, high-density lipoprotein (HDL) cholesterol, and glucose were measured using enzymatic colorimetric assays (Cobas 8000, Roche Diagnostics, Rotkreuz, Switzerland). Fasting glucose was defined as serum glucose levels obtained after a fasting period of at least 8 h. Non-HDL cholesterol was calculated by subtracting HDL from total cholesterol.

Insulin concentrations were determined using an electrochemiluminescence immunoassay (Cobas 8000, Roche Diagnostics, Rotkreuz, Switzerland). Insulin resistance was estimated using the Homeostatic Model Assessment for Insulin Resistance (HOMA-IR), calculated as fasting insulin (mU/L) multiplied by fasting glucose (mmol/L) divided by 22.5.

### 2.3. Liver Fat Quantification

Liver fat content was quantified using the CAP value derived from signals acquired with the FibroScan^®^ device (Echosense, Paris, France), a standardized and non-invasive modality with 87% sensitivity and 91% specificity for detecting hepatic steatosis [23,24]. The examinations were conducted with participants in the supine position, the right arm placed beneath the head, and the right leg crossed over the left to facilitate a slight leftward trunk rotation. The FibroScan^®^ M probe was positioned perpendicular to the skin surface within an intercostal space along the mid-axillary line. The xiphoid process served as an anatomical landmark to identify an suitable intercostal space overlying the center of the right hepatic lobe. Measurements were included in the final analysis only if the patient had fasted for at least three hours prior to the examination and a minimum of ten valid acquisitions were obtained. CAP values, expressed in decibels per meter (dB/m), were recorded within the device’s operational range of 100–400 dB/m. Manifest SLD was defined using the 90th percentile cutoff for CAP values based on a reference dataset [25].

### 2.4. Anthropometry

Body height and weight were assessed using calibrated scales, and body mass index (BMI) was calculated as weight (kg) divided by height squared (m^2^). Systolic and diastolic blood pressure were determined as the mean of three measurements taken on both the left and right arm taken after a 5 min seated rest, using an automated oscillometer device (OMRON M4-I, Omron Healthcare Co., Lake Forest, IL, USA). All anthropometric variables were transformed into age- and sex-specific z-scores based on German reference data [26,27].

### 2.5. Statistical Analysis

Characteristics of the study cohort are presented as mean ± standard deviation for continuous variables and as count (percentage) for categorial variables. Between-group differences were determined using the Student *t*-test for continuous variables and χ^2^ test for categorial variables, as appropriate.

Linear regression models were performed to determine the association between hs-CRP level and CAP value. Model 1 was unadjusted and model 2 was adjusted for sex and age. Ultimately, model 3 was adjusted for sex, age, HOMA-IR, non-HDL cholesterol, BMI z-score, SBP z-score. Additionally, simple and multivariable logistic regression analyses were performed with manifest SLD (i.e., CAP value above the 90th percentile of the reference dataset [25]) as the outcome variable and, as in the previous models, hs-CRP only (model 1), in addition to sex and age (model 2) and the aforementioned cardiometabolic risk factors (model 3) as predictor variables.

For all regression models, assumptions of independence of observations, normality of residuals, homoscedasticity, and linearity between dependent and independent variables were tested and met. In multivariable regression models, collinearity was verified using the variance inflation factor, which was <1.3 for all variables included. The analyses were performed using IBM SPSS Statistics version 29.0 (IBM, Armonk, NY, USA). All hypotheses tests were two-sided, and *p* values were considered statistically significant at *p* < 0.05.

## 3. Results

**Figure 1:**
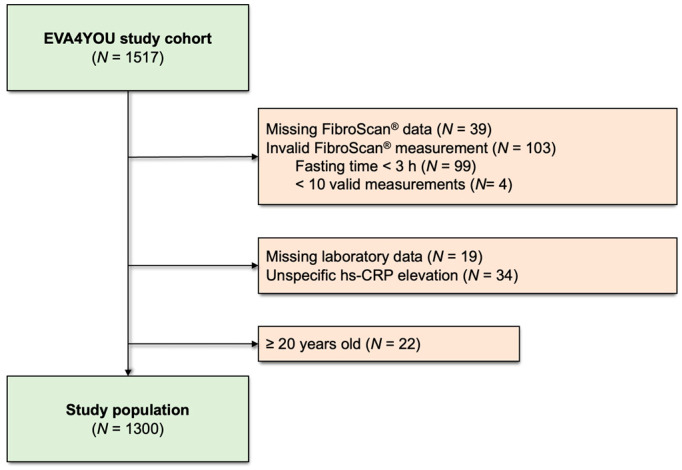
Study flow chart.

Of the 1517 participants in the EVA4YOU study, 142 were excluded from this analysis due to missing or invalid FibroScan^®^ measurements. In addition, 19 adolescents were excluded because of missing laboratory data, and 34 due to a non-specific hs-CRP elevation ≥ 10.0 mg/L. Another 22 were excluded due to an age of 20 years or older, resulting in a total cohort of 1300 participants (see flow chart in Figure 1).

As summarized in Table 1, the mean age of our study cohort was 17.2 ± 1.3 years, and 840 participants (64.6%) were female. Using the 90th percentile cutoff for CAP values of a reference dataset [25], 66 (5.1%) participants were classified as having manifest SLD. Compared to male participants, female adolescents had significantly lower CAP values and significantly higher hs-CRP levels (*p* < 0.001, both). Furthermore, there were significant sex differences in almost all cardiometabolic risk factors analyzed (except for HOMA-IR, *p* = 0.598). Detailed characteristics of the study population, including all analyzed variables, as well as a comparison of characteristics between participants with and without SLD, are presented in Tables S1 and S2 in the Supplementary Materials.

The linear regression models to determine the association between hs-CRP as a marker for systemic low-grade inflammation and CAP value are shown in Table 2. In model 1 (unadjusted), hs-CRP showed a significant positive correlation with the CAP value (*p* = 0.044), suggesting that with rising liver fat content, systemic low-grade inflammation increases. Adjustment for sex and age (model 2) further strengthens this correlation and its significance (*p* = 0.006).

However, further adjustment for cardiometabolic risk factors (HOMA-IR, non-HDL cholesterol, BMI z-score, SBP z-score) results in the loss of significance for the correlation between hs-CRP and CAP value (model 3; *p* = 0.419). It should be noted that the adjusted R^2^ is very low across all models (0.002 in model 1 and 0.052 in model 2 and 3), indicating that the models explain only a limited proportion of the variance in CAP.

Logistic regression analysis of hs-CRP in relation to manifest SLD—defined as CAP value ≥ 90th percentile of a reference dataset [25]—yielded similar results as the linear regression models shown in Table 2. The unadjusted model (model 1) showed an odds ratio (95% confidence interval) of 1.19 (1.06, 1.33). Similar estimates were observed after adjustment for sex and age (model 2) with an odds ratio of 1.20 (1.06, 1.36). However, the association was attenuated and no longer statistically significant after additional adjustment for components of the metabolic syndrome (model 3) with an odds ratio of 0.94 (0.78, 1.12; see Table S3 in Supplementary Materials).

## 4. Discussion

In our large, well characterized middle European cohort of adolescents we could correlate systemic low-grade inflammation, as measured by hs-CRP, to liver steatosis quantified by a non-invasive ultrasound method (CAP). Yet, after adjustment for components of the metabolic syndrome (HOMA-IR, non-HDL cholesterol, BMI z-score, and SBP z-score), this correlation appeared to be driven by the overall metabolic milieu rather than by liver specific inflammation.

Several previous studies have demonstrated that elevated hs-CRP levels are positively associated with the severity of SLD and with an increased risk of progression to steatohepatitis and liver fibrosis in adults [17,18,19,20], suggesting that systemic low-grade inflammation also reflects underlying regional hepatic inflammation. Our findings indicate that this seems not to be the case in adolescents. Instead, the shared metabolic background of the components of metabolic syndrome and its metabolic milieu likely accounts the observed correlation between CAP value and hs-CRP in the unadjusted model, as well as the subsequent loss of statistical significance after comprehensive adjustment in our adolescent cohort. hs-CRP is actually a well-established biomarker for systemic low-grade inflammation related to metabolic syndrome in adolescents and has been shown to be associated with its individual components, including insulin resistance, obesity, hypertension, and dyslipidemia [11,12,13].

The observed sex differences—namely lower CAP values and higher hs-CRP levels in females—are attributable to higher estrogen levels in females compared to males. Regarding hepatic steatosis, estrogens are assumed to exert a protective effect by enhancing the ability to adapt to metabolic responses to an excess of dietary lipids [28,29,30]. In the context of systemic low-grade inflammation, estrogens have been shown to promote the expression of inflammatory markers [31,32].

Although advanced liver steatosis and steatohepatitis have been documented in adolescents [4,33,34] and CAP values observed in our cohort indicate that 5.1% of our population exhibit manifest SLD, an important limitation of our study is the absence of histologic confirmation of SLD or steatohepatitis. Currently available non-invasive methods do not yet allow reliable differentiation between simple SLD and steatohepatitis [35]. A high prevalence of early-stage liver steatosis without clinically relevant hepatic inflammation might therefore have attenuated the associations between CAP and systemic low-grade inflammation in our adolescent cohort. Notably, our results remained unchanged when CAP was analyzed as a dichotomized outcome, further supporting the robustness of our results across different analytical approaches, although the low prevalence of manifest SLD limits the statistical power of the logistic regression analyses.

We assessed systemic inflammation solely using hs-CRP, a well-established and widely used biomarker, which, however, limits the interpretability of our findings. While several other inflammatory markers—such as vascular cell adhesion molecule-1 (VCAM-1), interleukin (IL)-6, IL-8, and various chemokines—have shown promise in distinguishing advanced fibrosis from milder disease stages in adults [36,37,38,39,40], these markers are rarely used in clinical practice and are associated with substantial costs.

Finally, it should be noted that this is a cross-sectional study, with all data collected at a single time point. As a result, only associations can be described, and no causal conclusions can be drawn. Future studies incorporating a broader panel of inflammatory markers, longitudinal designs, and, where feasible, histological or advanced imaging confirmation may help to further clarify the temporal and causal relationships between systemic low-grade inflammation and liver disease progression in adolescents.

Key strengths of our study include the large, community-based sample of adolescents of Central European descent, the high-quality assessment of cardiometabolic risk factors, and the standardized evaluation of liver steatosis using CAP. We cannot rule out that associations might be different in non-European genetic backgrounds.

## 5. Conclusions

In conclusion, our findings indicate that systemic low-grade inflammation is associated with the extent of liver steatosis in adolescence; however, this association is largely attributable to components of the metabolic syndrome and therefore appears to be driven by the metabolic milieu rather than by liver-specific inflammation. Consequently, our data suggest that hs-CRP does not serve as an independent risk factor for increased liver fat content in adolescents with simple steatosis.

## Figures and Tables

**Table 1:** Characteristics of the study population.

	All *N* = 1300 (100%)	Males *N* = 460 (35.4%)	Females *N* = 840 (64.6%)	*p* Value
Demographics				
Age, y	17.2 ± 1.3	17.3 ± 1.3	17.2 ± 1.3	0.255
Liver fat content				
CAP, dB/m	187.6 ± 39.7	198.7 ± 37.4	181.5 ± 39.6	**<0.001**
CAP ≥ 90th percentile ^a^	66 (5.1%)	32 (7.0%)	34 (4.0%)	**0.022 ^b^**
Systemic low-grade inflammation				
hs-CRP, mg/L	1.03 ± 1.63	0.77 ± 1.38	1.18 ± 1.74	**<0.001**
Cardiometabolic risk factors				
HOMA-IR, mU × mmol	2.6 ± 1.5	2.6 ± 1.5	2.6 ± 1.5	0.598
Non-HDL cholesterol, mmol/L	2.6 ± 0.7	2.5 ± 0.6	2.7 ± 0.7	**<0.001**
BMI, z-score	−0.068 ± 1.019	0.036 ± 0.962	−0.125 ± 1.045	**0.006**
SBP, z-score	0.978 ± 1.073	0.821 ± 0.984	1.063 ± 1.109	**<0.001**

Values are given as mean ± standard deviation or count (%). Between-group differences were determined using Student *t*-test (without adjustment for multiple comparisons), if not otherwise specified. Statistically significant *p* values are highlighted in bold. Missing data were <2% for all assessed parameters except for HOMA-IR (4.9%) and non-HDL cholesterol (8.6%). CAP, controlled attenuation parameter; hs-CRP, high-sensitivity C-reactive protein; HOMA-IR, Homeostatic Model Assessment for Insulin Resistance; HDL, high-density lipoprotein; BMI, body mass index; and SBP, systolic blood pressure. ^a^ Threshold for manifest steatotic liver disease; calculated using a reference data set [25]. ^b^ χ^2^ test.

**Table 2:** Association between hs-CRP and CAP value.

	Model 1 (Adjusted R^2^ = 0.002)		Model 2 (Adjusted R^2^ = 0.052)		Model 3 (Adjusted R^2^ = 0.052)	
	Regression coefficient (95% CI)	*p* value	Regression coefficient (95% CI)	*p* value	Regression coefficient (95% CI)	*p* value
hs-CRP, mg/L	1.35 (0.03, 2.68)	**0.044**	1.84 (0.53, 3.14)	**0.006**	−0.55 (−1.89, 0.79)	0.419

Linear regression models. Regression coefficients indicate the change in CAP (dB/m) per 1 mg/L increase in hs-CRP. Statistically significant *p* values are highlighted in bold. Model 1 is unadjusted. Model 2 with adjustment for sex and age. Model 3 included further adjustments for Homeostatic Model Assessment for Insulin Resistance, non-high-density lipoprotein cholesterol, body mass index z-score, and systolic blood pressure z-score. hs-CRP, high-sensitivity C-reactive protein; CAP, controlled attenuation parameter; and CI, confidence interval.

## Data Availability

The raw data supporting the conclusions of this article will be made available by the authors on request.

## Supplementary Materials

The following supporting information can be downloaded at: https://www.mdpi.com/article/10.3390/metabo16020108/s1, Table S1: Detailed characteristics of the study population; Table S2: Characteristics of the study population (based on the presence or absence of SLD); Table S3: hs-CRP as a predictor for SLD (i.e., CAP value ≥ 90th percentile of a reference dataset [25]).

## Abbreviations

The following abbreviations are used in this manuscript:

BMI	Body mass index
CAP	Controlled attenuation parameter
dB/m	Decibels per meter
EVA4YOU	Early Vascular Ageing in the YOUth
HDL	High-density lipoprotein
HOMA-IR	Homeostatic Model Assessment for Insulin Resistance
hs-CRP	High sensitivity C-reactive protein
MASLD	Metabolic dysfunction-associated steatotic liver disease
SLD	Steatotic liver disease

## References

[1] Targher G., Valenti L., Byrne C.D. (2025). Metabolic Dysfunction-Associated Steatotic Liver Disease. N. Engl. J. Med..

[2] Rinella M.E., Lazarus J.V., Ratziu V., Francque S.M., Sanyal A.J., Kanwal F., Romero D., Abdelmalek M.F., Anstee Q.M., Arab J.P. (2023). A multisociety Delphi consensus statement on new fatty liver disease nomenclature. J. Hepatol..

[3] Nairz J., Messner A., Kiechl S.J., Winder B., Hochmayr C., Granna J., Egger A.E., Griesmacher A., Geiger R., Knoflach M. (2024). Prevalence of metabolic dysfunction-associated steatotic liver disease (MASLD) and its association with arterial stiffness in adolescents: Results from the EVA4YOU study. PLoS ONE.

[4] Xanthakos S.A., Ibrahim S.H., Adams K., Kohli R., Sathya P., Sundaram S., Vos M.B., Dhawan A., Caprio S., Behling C.A. (2025). AASLD Practice Statement on the evaluation and management of metabolic dysfunction-associated steatotic liver disease in children. Hepatology.

[5] Devasia A.G., Ramasamy A., Leo C.H. (2025). Current Therapeutic Landscape for Metabolic Dysfunction-Associated Steatohepatitis. Int. J. Mol. Sci..

[6] Kuchay M.S., Choudhary N.S., Ramos-Molina B. (2025). Pathophysiological underpinnings of metabolic dysfunction-associated steatotic liver disease. Am. J. Physiol. Cell Physiol..

[7] Israelsen M., Francque S., Tsochatzis E.A., Krag A. (2024). Steatotic liver disease. Lancet.

[8] Wang M., Li L., Xu Y., Du J., Ling C. (2022). Roles of hepatic stellate cells in NAFLD: From the perspective of inflammation and fibrosis. Front. Pharmacol..

[9] Moutachakkir M., Lamrani Hanchi A., Baraou A., Boukhira A., Chellak S. (2017). Immunoanalytical characteristics of C-reactive protein and high sensitivity C-reactive protein. Ann. Biol. Clin..

[10] Pathak A., Agrawal A. (2019). Evolution of C-Reactive Protein. Front. Immunol..

[11] Ford E.S., Ajani U.A., Mokdad A.H., Examination N.H.A.N. (2005). The metabolic syndrome and concentrations of C-reactive protein among U.S. youth. Diabetes Care.

[12] de Ferranti S.D., Gauvreau K., Ludwig D.S., Newburger J.W., Rifai N. (2006). Inflammation and changes in metabolic syndrome abnormalities in US adolescents: Findings from the 1988–1994 and 1999–2000 National Health and Nutrition Examination Surveys. Clin. Chem..

[13] de F. Rocha A.R., de S. Morais N., Priore S.E., do C. C. Franceschini S. (2022). Inflammatory Biomarkers and Components of Metabolic Syndrome in Adolescents: A Systematic Review. Inflammation.

[14] Amezcua-Castillo E., González-Pacheco H., Sáenz-San Martín A., Méndez-Ocampo P., Gutierrez-Moctezuma I., Massó F., Sierra-Lara D., Springall R., Rodríguez E., Arias-Mendoza A. (2023). C-Reactive Protein: The Quintessential Marker of Systemic Inflammation in Coronary Artery Disease-Advancing toward Precision Medicine. Biomedicines.

[15] Chen L., Wang M., Yang C., Wang Y., Hou B. (2023). The role of high-sensitivity C-reactive protein serum levels in the prognosis for patients with stroke: A meta-analysis. Front. Neurol..

[16] Wijnstok N.J., Twisk J.W., Young I.S., Woodside J.V., McFarlane C., McEneny J., Hoekstra T., Murray L., Boreham C.A. (2010). Inflammation markers are associated with cardiovascular diseases risk in adolescents: The Young Hearts project 2000. J. Adolesc. Health.

[17] Okekunle A.P., Youn J., Song S., Chung G.E., Yang S.Y., Kim Y.S., Lee J.E. (2023). Predicted pro-inflammatory hs-CRP score and non-alcoholic fatty liver disease. Gastroenterol. Rep..

[18] Yoneda M., Mawatari H., Fujita K., Iida H., Yonemitsu K., Kato S., Takahashi H., Kirikoshi H., Inamori M., Nozaki Y. (2007). High-sensitivity C-reactive protein is an independent clinical feature of nonalcoholic steatohepatitis (NASH) and also of the severity of fibrosis in NASH. J. Gastroenterol..

[19] Jamialahmadi T., Bo S., Abbasifard M., Sathyapalan T., Jangjoo A., Moallem S.A., Almahmeed W., Ashari S., Johnston T.P., Sahebkar A. (2023). Association of C-reactive protein with histological, elastographic, and sonographic indices of non-alcoholic fatty liver disease in individuals with severe obesity. J. Health Popul. Nutr..

[20] Zhu C., Huang D., Ma H., Qian C., You H., Bu L., Qu S. (2022). High-Sensitive CRP Correlates with the Severity of Liver Steatosis and Fibrosis in Obese Patients with Metabolic Dysfunction Associated Fatty Liver Disease. Front. Endocrinol..

[21] Pearson T.A., Mensah G.A., Alexander R.W., Anderson J.L., Cannon R.O., Criqui M., Fadl Y.Y., Fortmann S.P., Hong Y., Myers G.L. (2003). Markers of inflammation and cardiovascular disease: Application to clinical and public health practice: A statement for healthcare professionals from the Centers for Disease Control and Prevention and the American Heart Association. Circulation.

[22] Myers G.L., Rifai N., Tracy R.P., Roberts W.L., Alexander R.W., Biasucci L.M., Catravas J.D., Cole T.G., Cooper G.R., Khan B.V. (2004). CDC/AHA Workshop on Markers of Inflammation and Cardiovascular Disease: Application to Clinical and Public Health Practice: Report from the laboratory science discussion group. Circulation.

[23] Karlas T., Petroff D., Sasso M., Fan J.G., Mi Y.Q., de Lédinghen V., Kumar M., Lupsor-Platon M., Han K.H., Cardoso A.C. (2017). Individual patient data meta-analysis of controlled attenuation parameter (CAP) technology for assessing steatosis. J. Hepatol..

[24] Pu K., Wang Y., Bai S., Wei H., Zhou Y., Fan J., Qiao L. (2019). Diagnostic accuracy of controlled attenuation parameter (CAP) as a non-invasive test for steatosis in suspected non-alcoholic fatty liver disease: A systematic review and meta-analysis. BMC Gastroenterol..

[25] Ramírez-Vélez R., García-Hermoso A., Correa-Rodríguez M., Izquierdo M. (2022). Defining values for controlled attenuation parameter and liver stiffness in youth without liver disease. Pediatr. Res..

[26] Kromeyer-Hauschild K., Wabitsch M., Kunze D., Geller F., Geiß H., Hesse V., von Hippel A., Jaeger U., Johnsen D., Korte W. (2001). Perzentile für den body-mass-index für das Kindes-und Jugendalter unter Heranziehung verschiedener deutscher Stichproben. Monatsschrift Kinderheilkd..

[27] Neuhauser H.K., Thamm M., Ellert U., Hense H.W., Rosario A.S. (2011). Blood pressure percentiles by age and height from nonoverweight children and adolescents in Germany. Pediatrics.

[28] Kur P., Kolasa-Wołosiuk A., Misiakiewicz-Has K., Wiszniewska B. (2020). Sex Hormone-Dependent Physiology and Diseases of Liver. Int. J. Environ. Res. Public Health.

[29] Della Torre S., Mitro N., Meda C., Lolli F., Pedretti S., Barcella M., Ottobrini L., Metzger D., Caruso D., Maggi A. (2018). Short-Term Fasting Reveals Amino Acid Metabolism as a Major Sex-Discriminating Factor in the Liver. Cell Metab..

[30] Meda C., Barone M., Mitro N., Lolli F., Pedretti S., Caruso D., Maggi A., Della Torre S. (2020). Hepatic ERα accounts for sex differences in the ability to cope with an excess of dietary lipids. Mol. Metab..

[31] Störk S., Bots M.L., Grobbee D.E., van der Schouw Y.T. (2008). Endogenous sex hormones and C-reactive protein in healthy postmenopausal women. J. Intern. Med..

[32] Conroy S.M., Neilson H.K., O’Reilly R., Woolcott C.G., Stanczyk F.Z., Courneya K.S., Friedenreich C.M. (2017). Associations between postmenopausal endogenous sex hormones and C-reactive protein: A clearer picture with regional adiposity adjustment?. Menopause.

[33] Souza M., Khalil S.M., de Oliveira F.D. (2025). Meta-analysis: Histological severity of biopsy-proven metabolic dysfunction-associated steatotic liver disease in pediatric patients. J. Pediatr. Gastroenterol. Nutr..

[34] Diehl A.M., Day C. (2017). Cause, Pathogenesis, and Treatment of Nonalcoholic Steatohepatitis. N. Engl. J. Med..

[35] Sheka A.C., Adeyi O., Thompson J., Hameed B., Crawford P.A., Ikramuddin S. (2020). Nonalcoholic Steatohepatitis: A Review. JAMA.

[36] Haukeland J.W., Damås J.K., Konopski Z., Løberg E.M., Haaland T., Goverud I., Torjesen P.A., Birkeland K., Bjøro K., Aukrust P. (2006). Systemic inflammation in nonalcoholic fatty liver disease is characterized by elevated levels of CCL2. J. Hepatol..

[37] Kar S., Paglialunga S., Jaycox S.H., Islam R., Paredes A.H. (2019). Assay validation and clinical performance of chronic inflammatory and chemokine biomarkers of NASH fibrosis. PLoS ONE.

[38] Bocsan I.C., Milaciu M.V., Pop R.M., Vesa S.C., Ciumarnean L., Matei D.M., Buzoianu A.D. (2017). Cytokines Genotype-Phenotype Correlation in Nonalcoholic Steatohepatitis. Oxid. Med. Cell. Longev..

[39] Auguet T., Bertran L., Binetti J., Aguilar C., Martínez S., Sabench F., Lopez-Dupla J.M., Porras J.A., Riesco D., Del Castillo D. (2020). Relationship between IL-8 Circulating Levels and TLR2 Hepatic Expression in Women with Morbid Obesity and Nonalcoholic Steatohepatitis. Int. J. Mol. Sci..

[40] Baltieri L., Chaim E.A., Chaim F.D.M., Utrini M.P., Gestic M.A., Cazzo E. (2018). Correlation Between Nonalcoholic Fatty Liver Disease Features and Levels of Adipokines and Inflammatory Cytokines Among Morbidly Obese Individuals. Arq. Gastroenterol..

