# Supplementary material for: hs-CRP as a Marker of Systemic Low-Grade Inflammation Is Not Associated with Steatotic Liver Disease in Adolescents: Insights from the EVA4YOU Study
Source: Metabolites. 2026 Feb 3;16(2):108. doi: 10.3390/metabo16020108 (PMC12942168; doi:10.3390/metabo16020108)
Supplement: Supplementary file 1 [file metabolites-16-00108-s001.zip › Table S1.pdf]

Table S1. Detailed characteristics of the study population.

	All $N = 1300$ (100%)	Males $N = 460$ (35.4%)	Females $N = 840$ (64.6%)	P value
**Demographics**				
Age, y	17.2 ± 1.3	17.3 ± 1.3	17.2 ± 1.3	0.255
**Liver fat content**				
CAP, dB/m	187.6 ± 39.7	198.7 ± 37.4	181.5 ± 39.6	**<0.001**
CAP ≥ 90th percentile [a]	66 (5.1%)	32 (7.0%)	34 (4.0%)	**0.022** [b]
**Systemic low-grade inflammation**				
hs-CRP, mg/L	1.03 ± 1.63	0.77 ± 1.38	1.18 ± 1.74	**<0.001**
**Cardiometabolic risk factors**				
Fasting glucose, mmol/L	4.2 ± 0.6	4.5 ± 0.6	4.1 ± 0.5	**<0.001**
Insulin, mU/L	13.7 ± 6.9	13.1 ± 6.3	14.0 ± 7.2	**0.049**
HOMA-IR, mU × mmol	2.6 ± 1.5	2.6 ± 1.5	2.6 ± 1.5	0.598
Total cholesterol, mmol/L	4.1 ± 0.7	3.8 ± 0.6	4.3 ± 0.8	**<0.001**
HDL cholesterol, mmol/L	1.5 ± 0.3	1.3 ± 0.3	1.6 ± 0.3	**<0.001**
Non-HDL cholesterol, mmol/L	2.6 ± 0.7	2.5 ± 0.6	2.7 ± 0.7	**<0.001**
BMI, kg/m^2	22.2 ± 3.5	22.4 ± 3.4	22.0 ± 3.6	**0.028**
BMI, z-score	-0.068 ± 1.019	0.036 ± 0.962	-0.125 ± 1.045	**0.006**
SBP, mmHg	127 ± 11	132 ± 11	124 ± 11	**<0.001**
SBP, z-score	0.978 ± 1.073	0.821 ± 0.984	1.063 ± 1.109	**<0.001**
DBP, mmHg	75 ± 8	73 ± 7	76 ± 8	**<0.001**
DBP, z-score	0.536 ± 1.053	0.165 ± 0.961	0.739 ± 1.046	**<0.001**

Values are given as mean ± standard deviation or count (%). Between-group differences were determined using Student t test (without adjustment for multiple comparisons), if not otherwise specified. Missing data were < 2% for all assessed parameters except for HOMA-IR (4.9%), insulin (4.9%), and non-HDL cholesterol (8.6%).

CAP, controlled attenuation parameter; hs-CRP, high-sensitivity C-reactive protein; HOMA-IR, Homeostatic Model Assessment for Insulin Resistance; HDL, high-density lipoprotein; BMI, body mass index; SBP, systolic blood pressure; and DBP, diastolic blood pressure.
[a] Threshold for manifest steatotic liver disease; calculated using a reference data set [25].
[b] χ^2 test.
