# Supplementary material for: hs-CRP as a Marker of Systemic Low-Grade Inflammation Is Not Associated with Steatotic Liver Disease in Adolescents: Insights from the EVA4YOU Study
Source: Metabolites. 2026 Feb 3;16(2):108. doi: 10.3390/metabo16020108 (PMC12942168; doi:10.3390/metabo16020108)
Supplement: Supplementary file 1 [file metabolites-16-00108-s001.zip › Table S2.pdf]

Table S2. Characteristics of the study population (based on the presence or absence of SLD).

	All<br>*N* = 1300<br>(100%)	SLD [a]<br>*N* = 66<br>(5.1%)	no SLD<br>*N* = 1234<br>(64.6%)	P value
**Demographics**				
Age, y	17.2 ± 1.3	17.9 ± 1.2	17.2 ± 1.3	**<0.001**
Sex, male	460 (35.4%)	32 (48.5%)	428 (34.7%)	**0.022** [b]
**Systemic low-grade inflammation**				
hs-CRP, mg/L	1.03 ± 1.63	1.60 ± 2.05	1.00 ± 1.60	**0.022**
**Cardiometabolic risk factors**				
Fasting glucose, mmol/L	4.2 ± 0.6	4.5 ± 0.6	4.2 ± 0.6	**<0.001**
Insulin, mU/L	13.7 ± 6.9	20.7 ± 9.7	13.3 ± 6.6	**<0.001**
HOMA-IR, mU × mmol	2.6 ± 1.5	4.2 ± 2.2	2.5 ± 1.4	**<0.001**
Total cholesterol, mmol/L	4.1 ± 0.7	4.1 ± 0.7	4.1 ± 0.8	0.960
HDL cholesterol, mmol/L	1.5 ± 0.3	1.3 ± 0.3	1.5 ± 0.3	**<0.001**
Non-HDL cholesterol, mmol/L	2.6 ± 0.7	2.8 ± 0.7	2.6 ± 0.7	0.073
BMI, kg/m^2	22.2 ± 3.5	28.1 ± 4.2	21.8 ± 3.2	**<0.001**
BMI, z-score	-0.068 ± 1.019	1.274 ± 0.754	-0.140 ± 0.981	**<0.001**
SBP, mmHg	127 ± 11	132 ± 12	127 ± 11	**<0.001**
SBP, z-score	0.978 ± 1.073	1.201 ± 1.161	0.966 ± 1.067	0.082
DBP, mmHg	75 ± 8	77 ± 8	74 ± 8	**0.026**
DBP, z-score	0.536 ± 1.053	0.662 ± 1.116	0.529 ± 1.049	0.317

Values are given as mean ± standard deviation or count (%). Between-group differences were determined using Student t test (without adjustment for multiple comparisons), if not otherwise specified. Missing data were < 2% for all assessed parameters except for HOMA-IR (4.9%), insulin (4.9%), and non-HDL cholesterol (8.6%).

SLD, steatotic liver disease; hs-CRP, high-sensitivity C-reactive protein; HOMA-IR, Homeostatic Model Assessment for Insulin Resistance; HDL, high-density lipoprotein; BMI, body mass index; SBP, systolic blood pressure; DBP, diastolic blood pressure; and CAP, controlled attenuation parameter.

[a] Defined as CAP value ≥ 90th percentile of a reference dataset [25].

[b] χ^2 test.
