# Supplementary material for: hs-CRP as a Marker of Systemic Low-Grade Inflammation Is Not Associated with Steatotic Liver Disease in Adolescents: Insights from the EVA4YOU Study
Source: Metabolites. 2026 Feb 3;16(2):108. doi: 10.3390/metabo16020108 (PMC12942168; doi:10.3390/metabo16020108)
Supplement: Supplementary file 1 [file metabolites-16-00108-s001.zip › Table S3.pdf]

Table S3. hs-CRP as a predictor for SLD (i.e., CAP value ≥ 90th percentile of a reference dataset [25]).

	Model 1 (Nagelkerke R^2 = 0.016)			Model 2 (Nagelkerke R^2 = 0.070)			Model 3 (Nagelkerke R^2 = 0.382)		
	b (SE)	P value	OR (95% CI)	*b* (SE)	P value	OR (95% CI)	*b* (SE)	P value	OR (95% CI)
hs-CRP, mg/L	0.17 (0.06)	**0.004**	1.19 (1.06, 1.33)	0.18 (0.06)	**0.003**	1.20 (1.06, 1.36)	-0.07 (0.09)	0.463	0.94 (0.78, 1.12)

Logistic regression models. Model 1 is unadjusted. Model 2 with adjustment for sex and age. Model 3 with adjustment for Homeostatic Model Assessment for Insulin Resistance, non-high-density lipoprotein cholesterol, body mass index z-score, and systolic blood pressure z-score.

hs-CRP, high-sensitive C-reactive protein; SLD, steatotic liver disease; CAP, controlled attenuation parameter; SE, standard error; OR, odds ratio; and CI, confidence interval.
